# A three-dimensional intelligent engineering management and control system for the construction of a long-span valve hall project based on a microservice architecture

**DOI:** 10.1371/journal.pone.0261012

**Published:** 2021-12-03

**Authors:** Qinghe Zeng, WeiHua Ming, Jin Luo, SongAn Zhang, Wei Hu, Zhen Liu, CuiYing Zhou

**Affiliations:** 1 China Southern Power Grid Ehv Power Transmission Company, Guangzhou, China; 2 School of Civil Engineering, Sun Yat-sen University, Guangzhou, China; Al Mansour University College-Baghdad-Iraq, IRAQ

## Abstract

Three-dimensional intelligent engineering management and control systems (EMCS) based on the browser/server (B/S) model are an important part of intelligent engineering development. These systems are used for solving the difficulties encountered in engineering management with frequent cross-specialties and are vital tools for data exchange and service sharing among multiple departments. Currently, most engineering management and control systems are based on service-oriented architectures (SOAs). The integration mechanism and high coupling of SOAs leads to the reduction in system expansibility, service quality and service safety of the engineering system, making it difficult for these architectures to serve the construction of long-span valve hall engineering. To address these concerns, the management and application technology of the multidisciplinary data of valve hall engineering based on a microservice architecture (MSA) is proposed to improve the management efficiency of engineering data. A 3D integration modeling method for valve hall engineering structures and geological environments is proposed to establish the topological association between engineering structures and geological environments, without increasing the amount of model data required. A 3D intelligent engineering management and control technology for the entire process of the construction of long-span valve hall engineering is proposed, which realizes the entire process simulation and control of engineering construction based on WebGL technology. Accordingly, a three-dimensional intelligent engineering management and control system for the entire construction process of a long-span valve hall project in Southeast China is established, which can effectively manage and apply the data, display and analyze the three-dimensional model, and control and make decisions regarding the construction schedule. This study provides support for the construction of "smart engineering", promotes information communication and transmission between different project units, and speeds up the transformation from traditional construction management relying on drawings to three-dimensional intelligent construction management based on cloud services.

## 1 Introduction

Long-distance high-voltage direct current (HVDC) transmission projects have significant advantages in terms of transmission stability and account for an increasing proportion of China’s power grid construction projects, and the corresponding converter station construction has also been increasing rapidly [1]. A valve hall structure is one of the key facilities in the construction of HVDC projects, and it is an indispensable structure in power grid systems. Therefore, the construction of valve halls is an important part of the construction of HVDC projects. With the rapid development of the Internet, engineering construction management integrated with information technology has become an inevitable trend [2, 3]. Construction data play an increasingly important role in overall construction projects and thus the realization of construction information, efficient data sharing, and control visualization are important for ensuring the safety, quality, and progress of construction projects [4, 5].

A C/S structure engineering management system based on a building information model (BIM) has become an important means for improving information generation and management in the design process [6–8]. With the rapid development of network technology, traditional engineering management systems based on C/S structures have gradually been developed based on B/S or hybrid structures owing to their shortcomings, such as their inability to achieve cross-platform and network sharing, high maintenance costs and inconvenient information acquisition. Matthews et al. studied the effectiveness of a BIM based on the cloud in real-time information transmission to support progress monitoring and management in the construction of reinforced concrete structures; examined the effectiveness of a cloud-based BIM for the real-time delivery of information to support the progress monitoring and management of the construction of a reinforced concrete (RC) structure using action-based research [8]. Yang adopted a combination of the B/S and C/S models to establish an irrigation information management system [9]. Luo designed an information management system for a regional cleaner production structure design based on the B/S structure [10]. However, existing engineering management and control systems are still based on SOAs. The integration mechanism and high coupling of SOAs cause engineering systems to reduce system expansibility, service quality and service safety [11, 12]. Traditional engineering management and control systems face difficulties in meeting the growing demand for engineering management services. Therefore, the study and design of the architecture of engineering management and control system platforms are urgently needed.

The construction of long-span valve hall projects is an important topic in the research field of engineering management and control systems. Valve hall projects are information-intensive, which means that it is crucial to be able to access accurate information at the right time and place for decision making and project schedules. Inadequate information provided during construction has been identified as a contributing factor to low productivity and rework, resulting in project delays and cost overruns [13]. With construction progress, the scale of engineering data has been increasing. The traditional safety decision-making management method is not sufficient to adapt to the transient, dynamic and variable nature of engineering [14]. Therefore, it is necessary to establish a database cluster based on MSA, study the classification and coding technology of the construction information of valve hall projects, and realize efficient updating, management and application of the data for the entire construction process to promote data sharing and service sharing across departments [15].

In view of the aforementioned problems, this study proposes three technical methods based on MSA: the management and application of multi-professional data, the 3D integration modeling method of engineering structures and geological environments, and the three-dimensional intelligent control of the entire construction process of the long-span valve hall project. Integrating these methods, a three-dimensional intelligent engineering management and control system for the construction of a long-span valve hall project is established, which is applied in a long-span valve hall project in Southeast China. It has been proven that this system has high expansibility, rationality and service reliability.

## 2 System design

### 2.1 Engineering data management and application

The engineering data management and application of a long-span valve hall based on MSA is the foundation of an engineering management and control system, which provides data support for corresponding services. When designing the system, the following two problems need to be addressed: a) a large amount of data and frequent updates lead to a reduction in the efficiency of storage, query and analysis, and b) the variety in the types of data leads to confusion in data management. Therefore, it is necessary to assign more data management tasks to the server, standardize the classification of data management on the internet side, and create a valve hall engineering database cluster based on the MSA [16].

Engineering data include design data, numerical simulation data, construction element data, progress data, monitoring data, etc. Data can be divided into different categories according to type: tabular data, spatial data, document video data and other file data. The principle of data classification is an important factor affects the efficiency of data management and applications.

**Fig 1:**
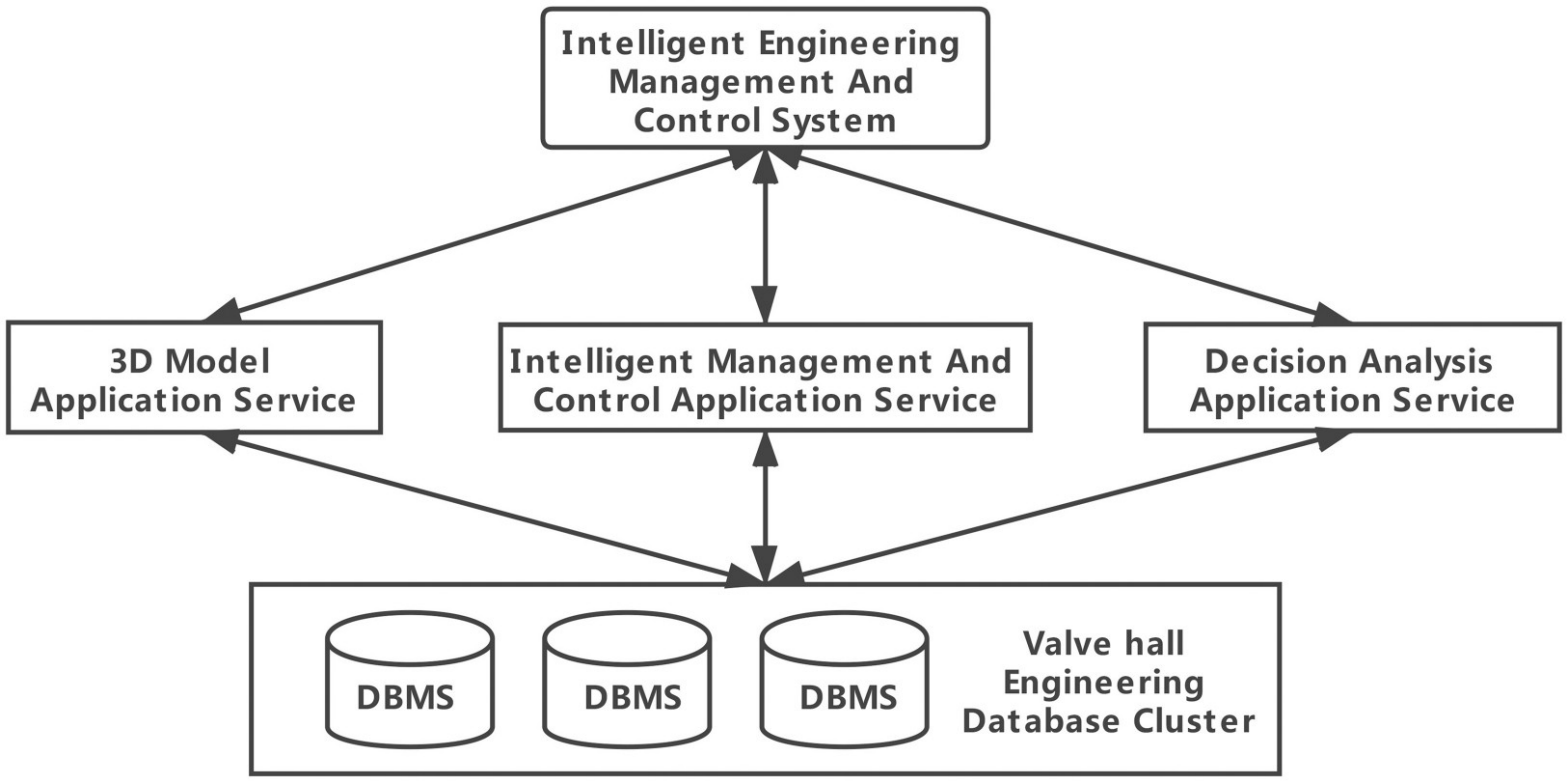
Database cluster service.

**Fig 2:**
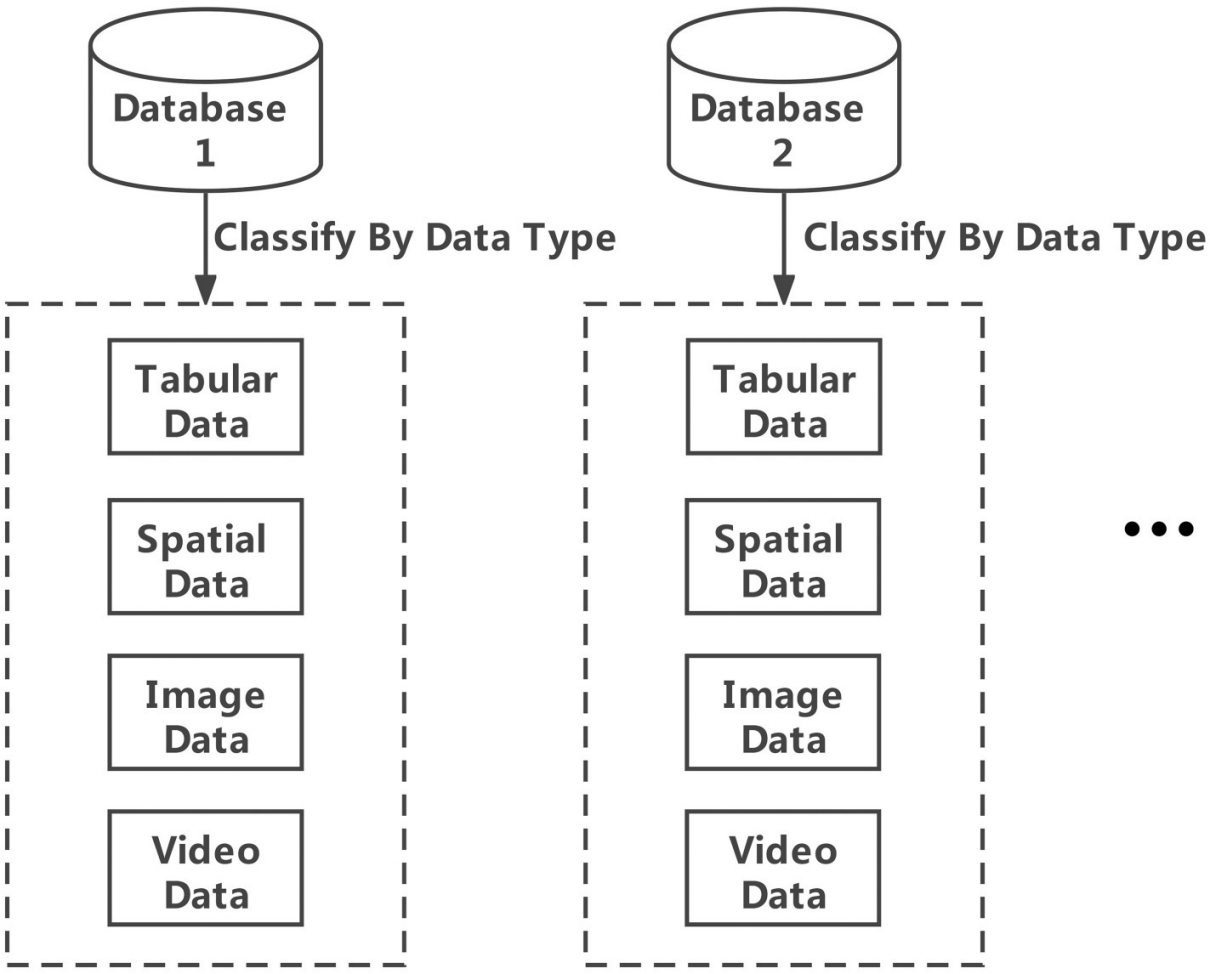
Data type classification diagram.

The traditional B/S structure of engineering management and control systems is based on SOAs. The SOA integration mechanism allows the system to support the core application services of the entire service platform with a single database [17, 18]. With the continuous updating of data and the increase in the number of data characteristics, the single database model has become bloated, and the management of engineering data has become increasingly difficult. At the same time, owing to the unique data structure and characteristics of each relevant department, it is impractical to establish a unified data standard that integrates all units or departments. Therefore, the intelligent engineering management and control system is divided into several independent application services, and the corresponding databases are created according to different application service functions. The engineering data of each database are classified according to the type. The database cluster service architecture of this system is illustrated in Fig 1. In a single database, the data are classified by type, as shown in Fig 2.

The convenient, effective and intelligent control of the lifecycle of a large engineering project is essentially the control of an enormous amount of information. With the development of engineering projects, the rapid expansion of engineering information has led to great challenges in terms of data transmission and management. The emergence of information coding technology has effectively solved the problem of information management and interaction in the construction stage. Information coding is the transformation of a symbol system representing things (or concepts) into another symbol system that is easy for computers or people to recognize and process. Moreover, information coding can improve the ability and speed of computer information processing and promote information exchange and data sharing for each service within the system. The consistency of information transmission and exchange between services is the premise and foundation of information sharing and interoperation between systems. To improve the network transmission speed of valve hall engineering data and ensure information sharing and exchange among various services within the system, a long-span valve hall engineering information classification and coding system is studied to realize the rapid management and analysis of data using an intelligent control system.

**Fig 3:**
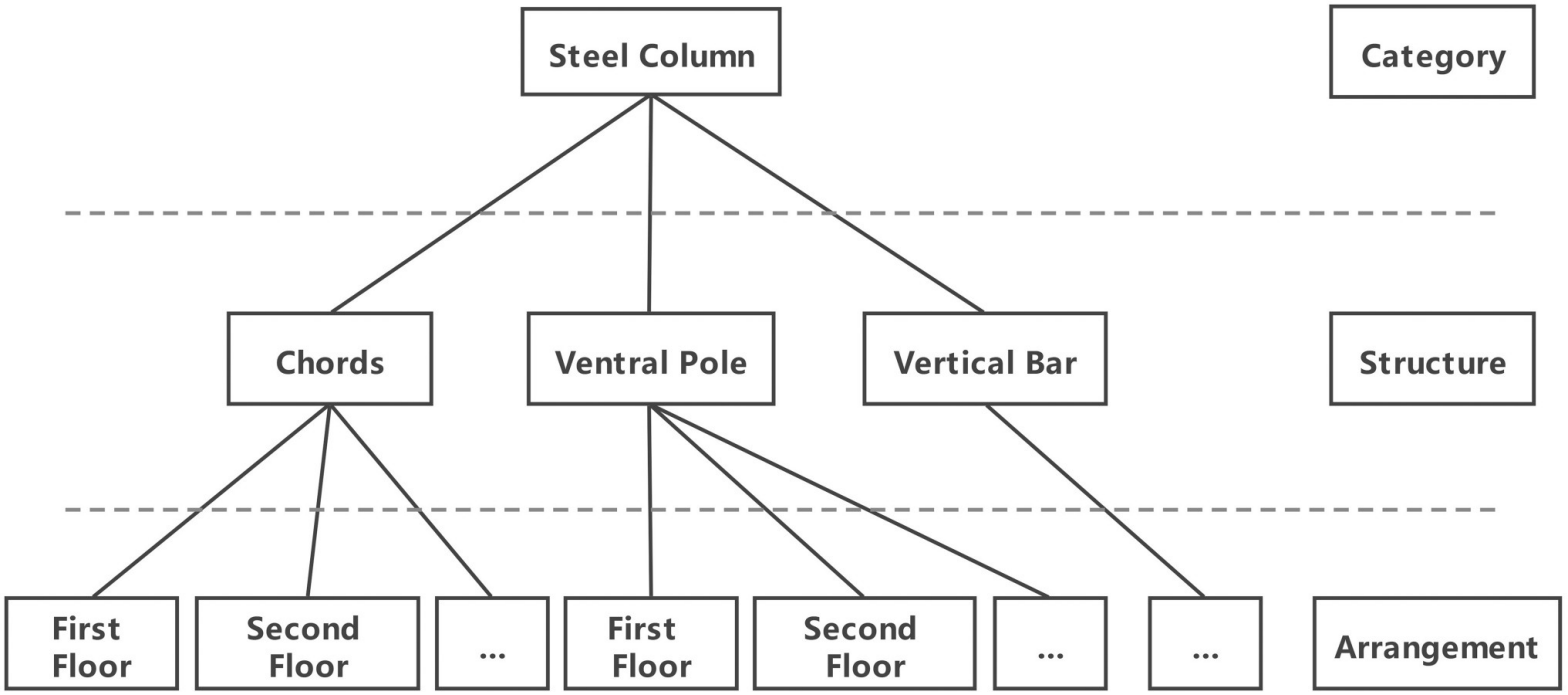
Classification diagram of steel structural members.

According to the characteristics of the steel structure in the project of a long-span valve hall, the line classification method is adopted, which can better reflect the logical relationships among the levels in each engineering component, at the same time, it conforms to the traditional method of processing information by hand and is convenient for computer processing. The classification of the steel structure members is shown in Fig 3.

**Fig 4:**
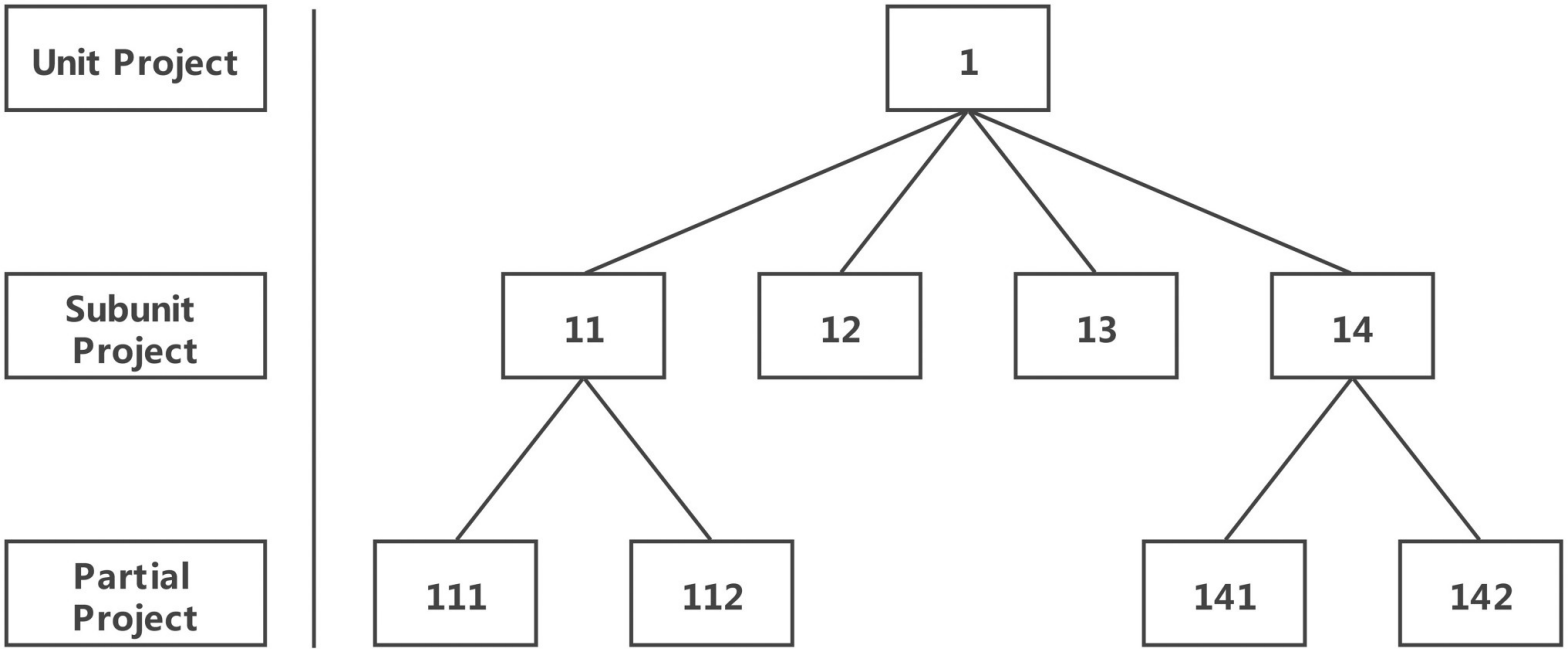
Structure relation code of valve hall engineering project.

The construction of a long-span valve hall involves many industries. In view of this situation, the project is divided by structural relationship characteristic codes, and the project information code is added before the component code so that each construction project stage can be fully understood in the service information exchange of the system, and the information management of each construction project can be effectively carried out. The structural relationship codes are shown in Fig 4.

**Fig 5:**
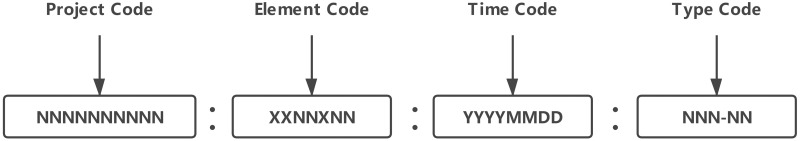
Information classification and coding system.

According to the actual situation of the project, both the work classification system and element classification system are quoted, and the coding system is set up in four layers, corresponding to the project code, element code, time code and work type code, as shown in Fig 5.

The project code is the division of construction projects and is obtained using engineering structure relation codes, which include unit, subunit, partial, subpartial, and subitem projects. Table 1 lists the partial unit and subunit project codes.

**Table 1:** Unit project code.

Project code	Project name
06	Flexible foundation and system structures
07	Valve hall and ancillary facilities
11	Fire protection system, structure and equipment installation
23	Equipment room

The element code is based on the Uniformat-II element concept. According to the engineering components and functional division, a part of the steel structure element code is listed in Table 2.

**Table 2:** Engineering element code for steel structure.

Element code			Element object
Element type code	Element type	Element code	
GZ	Steel column	GZ01S11	No. 1 chord of the first layer of No. 1 steel column
		GZ01S12	No. 2 chord on the first layer of No. 1 steel column
		GZ01H11	No. 1 transverse belly bar on the first layer
		GZ01X11	No. 1 diagonal belly pole on the first floor

The time code is used to describe the start time, end time or time period of the working section of the engineering construction component, the coding rules of which are listed in Table 3.

**Table 3:** Time code rule.

Time code	Time zone
20190000	During the period of 2019
20190100	During January 2019
20190101	January 1, 2019
20190101–20190501	January 1, 2019 to May 1, 2019

The work type code records how the construction personnel complete the work object and whether special information or circumstances need to be noted, including personnel codes, remark codes and other information.

### 2.2 Three-dimensional integrated modeling method of valve hall engineering structure and geological environment

At present, engineering management and control systems either have a single architectural structure model or geological model, and there is no model that integrates the two models. At the same time, owing to the limitation of the memory of the client machine, the display and analysis effect of the structure model or geological environment model on the browser is not satisfactory. Therefore, it is necessary to explore a method for realizing the integrated modeling of 3D valve hall engineering structures and geological environments without increasing the amount of model data to reduce the burden of browsers when displaying the model.

The topological association between the valve hall engineering structure and the geological environment was mainly recorded through the information of the contact nodes. The previous method redivides the units of the geological environment model to make the adjacent model nodes correspond to the engineering structure nodes one by one. The topological correlation established by this method requires significant subdivision work, which greatly increases the amount of model data. To address these problems, this study proposes a method for recording the contact surface. A curved surface unit was created between the geological environment model and the valve hall engineering structure, and the two sides of the curved surface were fitted with the geological environment model and the valve hall engineering structure model, respectively, and the associated nodes on both sides were recorded to keep track of the topological association between the geological environment model and the engineering structure.

**Fig 6:**
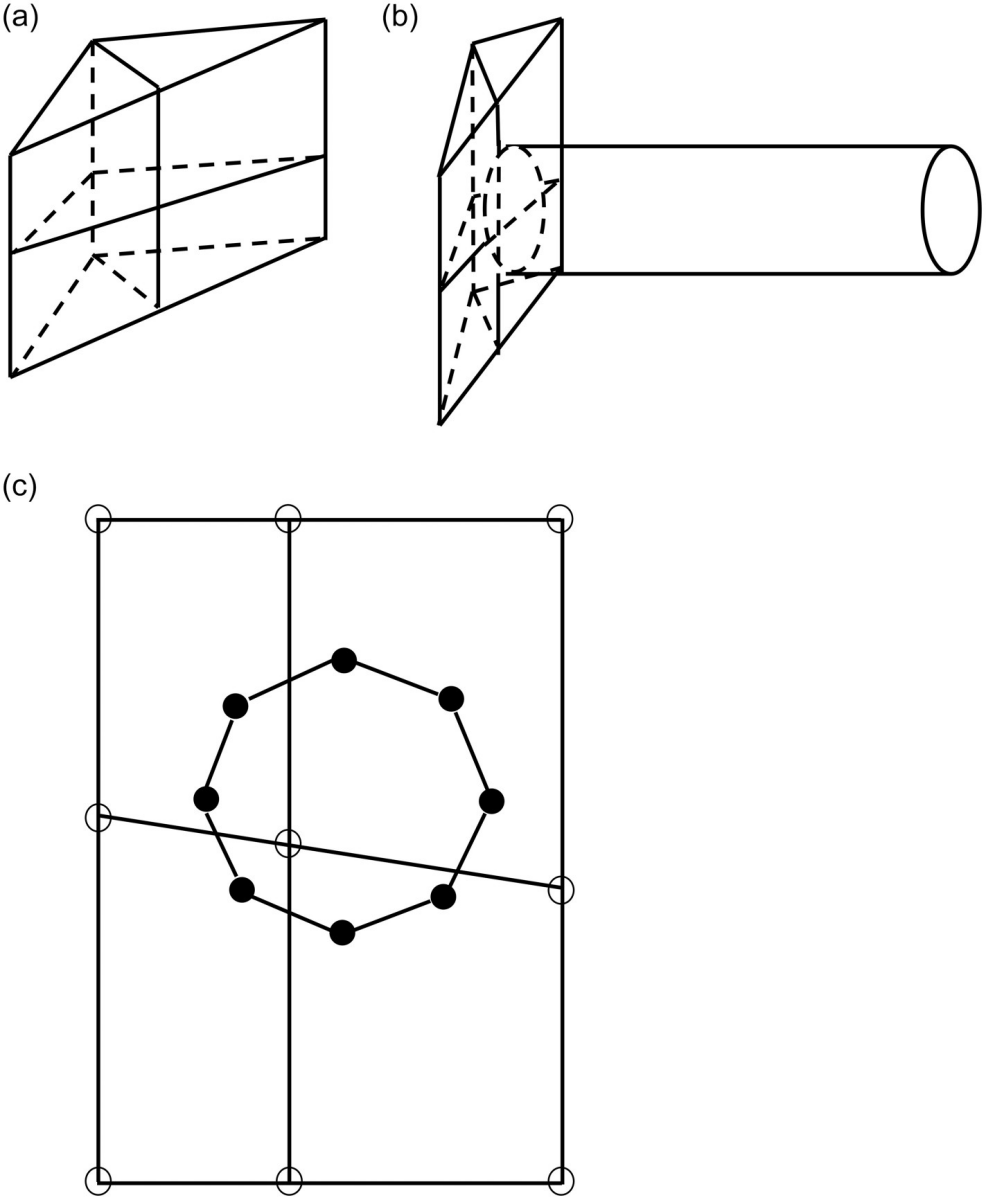
Diagram of contact between geological environment and engineering structure. (a) Geological environment model units; (b) Spatial position of the geological environment and engineering structure; (c) Schematic of the contact surface between the two.

Fig 6 shows the contact between the rock and the soil mass and a circular cross-section support. Fig 6(a)–6(c) present the geological environment model units, the spatial position of the geological environment and engineering structure, and the schematic diagram of the contact surface between the two, respectively. The surface records the nodes in contact with the engineering structure and those in contact with the geological model, which are represented by hollow circles and solid circles, respectively.

**Fig 7:**
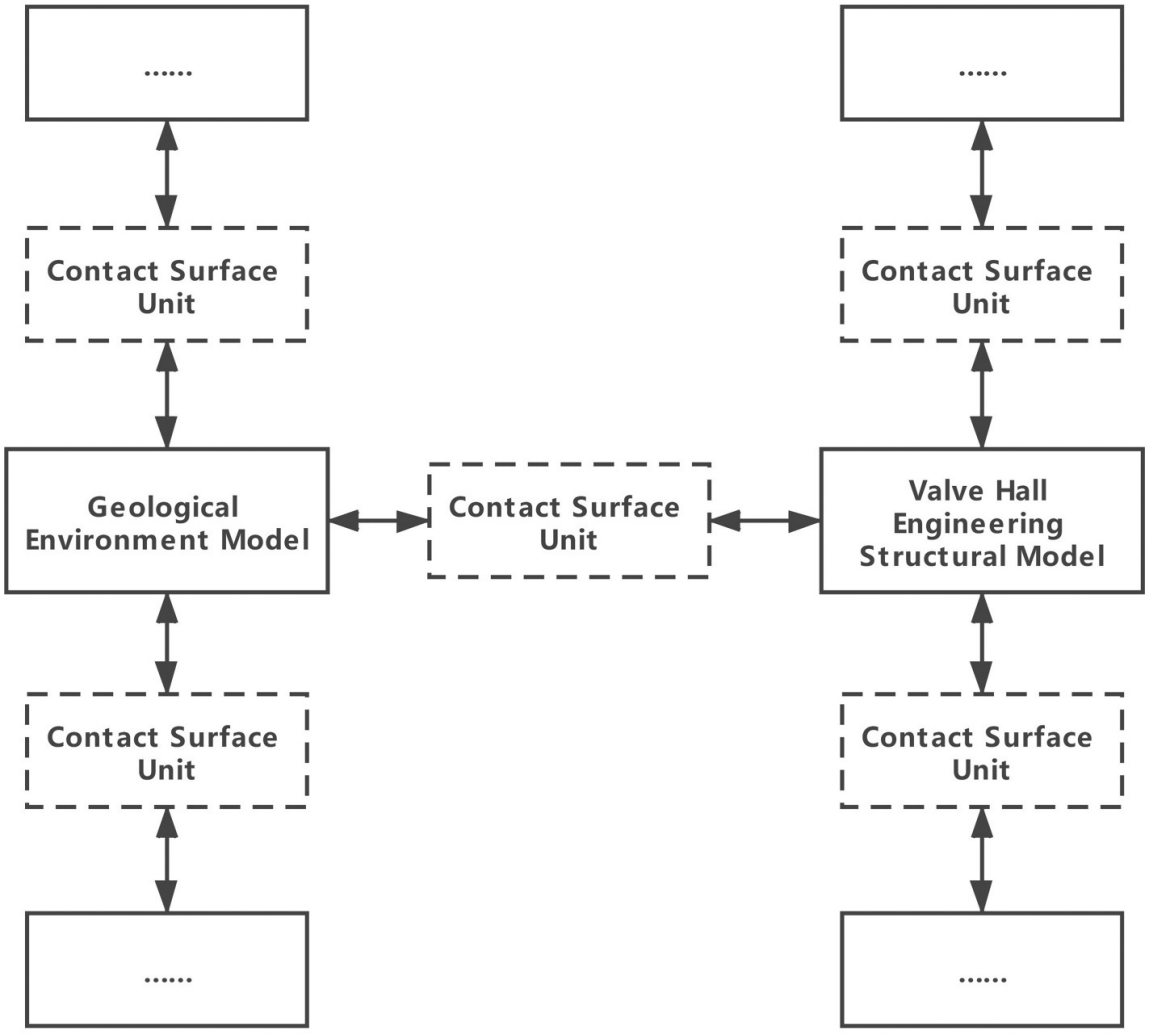
Topological relationship between geotechnical model and engineering structure.

Each geological environment model or engineering structure model unit records the information of multiple contact surfaces, each of which records the contact nodes of the corresponding geological environment model unit and valve hall engineering construction model on both sides, as shown in Fig 7.

Through this method, the topological correlation between the geological environment and the valve hall engineering structure can be established without significantly increasing the amount of model data. On this basis, the integrated modeling of the engineering structure and geological environment of the long-span valve hall based on MSA is realized using WebGL technology.

### 2.3 Three-dimensional intelligent control of the whole process construction

At present, there are three problems in the management and control of the construction of long-span valve hall projects. (1) The construction process is expressed by words and two-dimensional pictures, thus the understanding of this process is relatively abstract, which is not good for the control of the key process of this construction. (2) The existing construction management and control system can only display some figures and table data in the construction process, which cannot be displayed in real time through a combination of 3D models. (3) In the actual construction process, it is still necessary for professionals to manually compare the monitoring data with the numerical simulation data in the engineering design stage, which is costly, time-consuming and has a great impact on the timely control and quality of the construction schedule.

In view of the aforementioned situation, this study proposes the construction progress and quality control of super long-span valve halls based on MSA. Combined with the three-dimensional model, the complex structure of the valve hall becomes simpler in the management and control systems during construction. Through a continuous comparison of the construction plan, management personnel can accurately understand the construction progress and control the construction quality. Three-dimensional intelligent engineering management and control for the construction of long-span valve hall projects based on MSA focuses on the realization of construction progress and construction quality control through visualization technology.

#### 2.3.1 Construction progress control

The application of visualization technology in valve hall project schedule management is the concentrated embodiment of new information technology in construction projects. During construction, it is necessary to constantly grasp the implementation of the construction plan, compare and analyze the actual and planned situations, and take effective measures when necessary to make the project progress in accordance with the predetermined goal.

**Fig 8:**
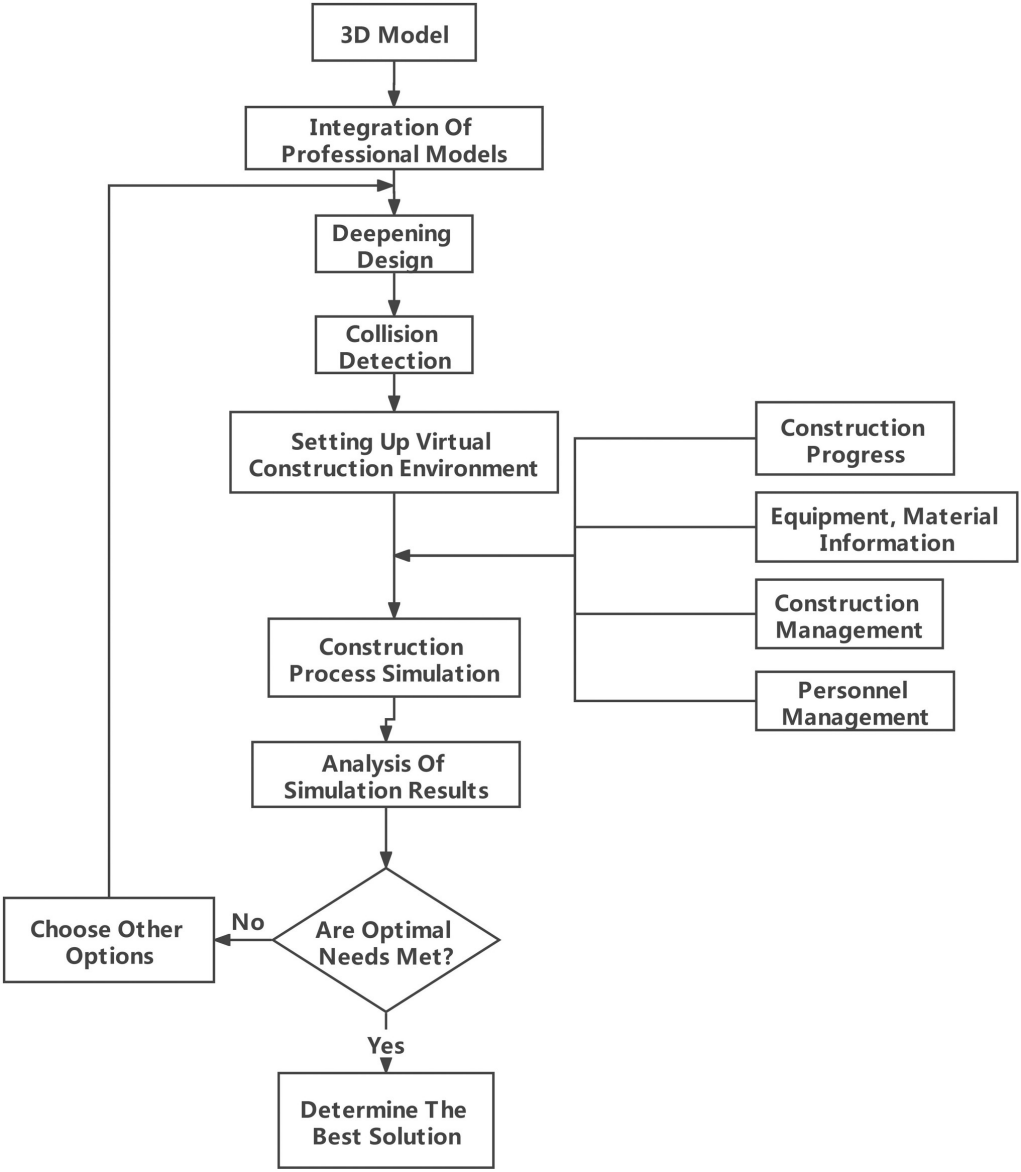
Construction progress control.

The three-dimensional intelligent engineering management and control systems for the construction of long-span valve hall projects based on MSA can realize the rapid updating of construction data and the expansion of independent services. Combined with the 3D visualization model, the construction scheme for each stage is simulated. Through the client browser, the project manager can accurately grasp the construction status in real time to ensure the orderly progress of each construction link. Fig 8 shows the simulation process of the complex construction scheme.

#### 2.3.2 Construction quality control

Numerical simulation data in the engineering design stage are important reference data for analyzing the structural stability, construction quality and safety. However, in the actual construction process, the monitoring and simulation data still need to be manually compared by professionals, which has a great impact on the timely management and control of the construction process.

**Fig 9:**
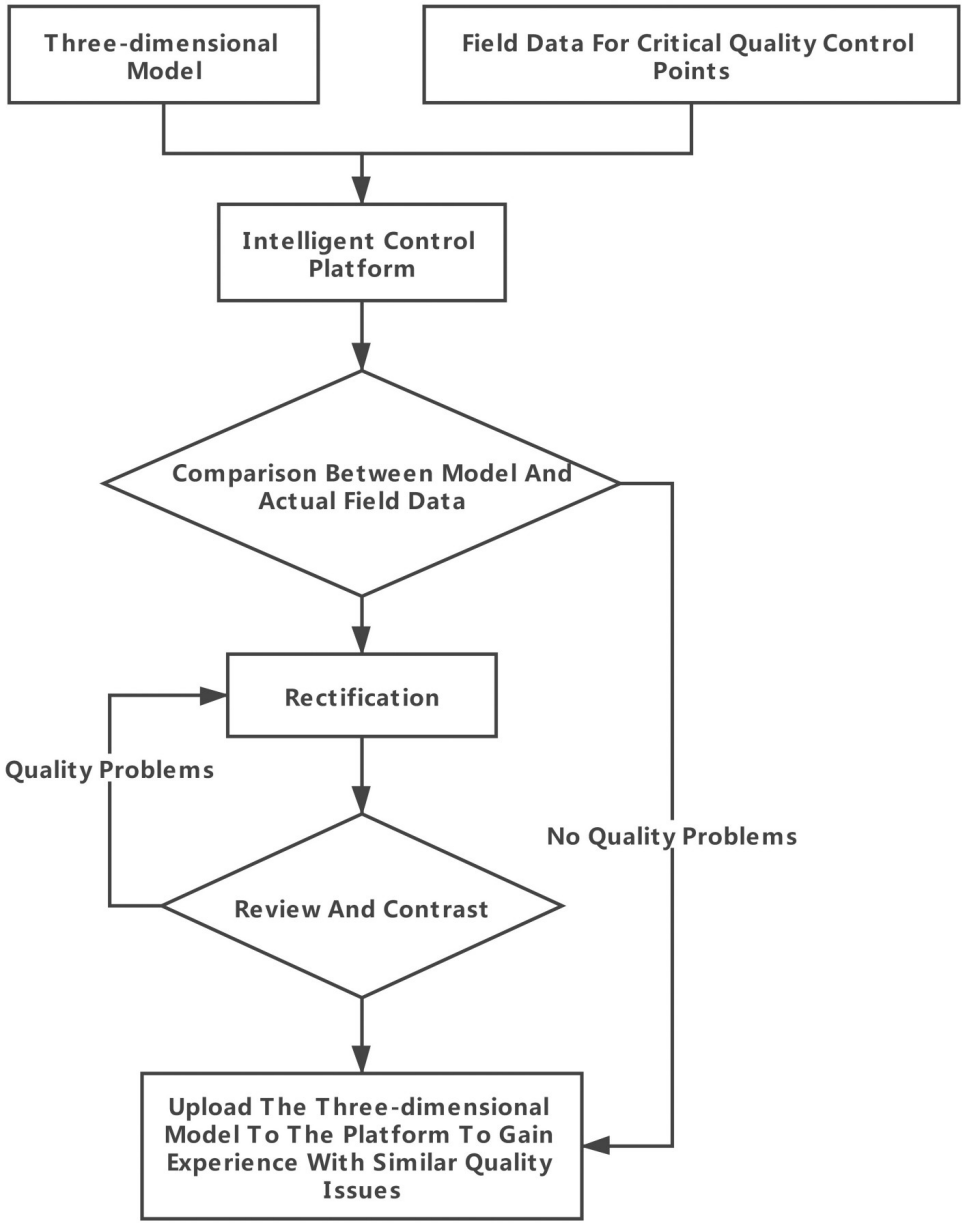
Quality control process.

To this end, this study proposes the quality control of the entire construction process of valve hall projects, establishes a numerical simulation database and construction monitoring database, and realizes the association of a 3D valve hall structure model with numerical simulation data and monitoring data through WebGL technology. Through real-time comparison of monitoring data and numerical simulation data, the quality problems in the construction process were effectively controlled. The main control process is illustrated in Fig 9.

## 3 Engineering application

**Fig 10:**
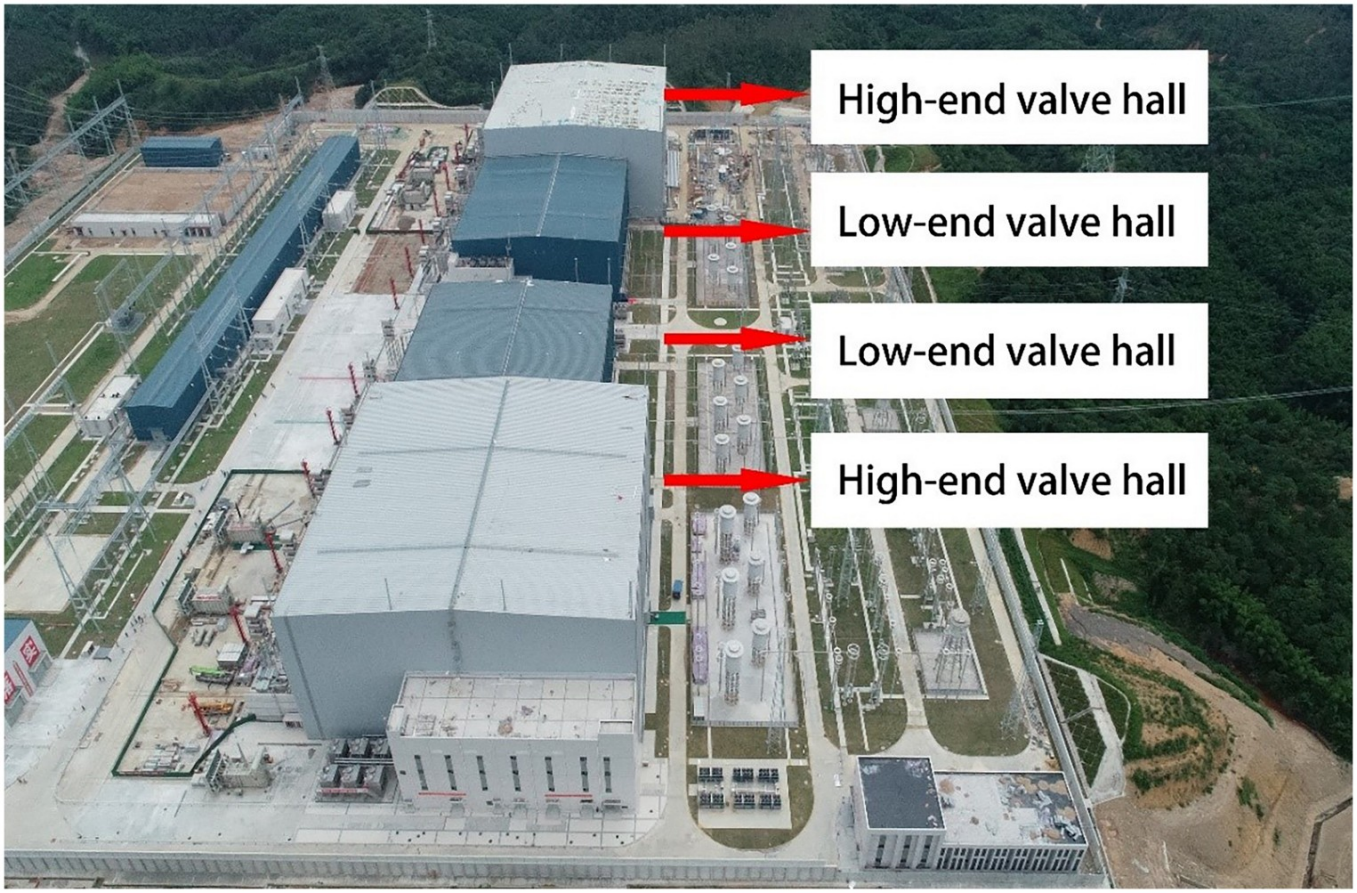
Image of the project site.

In this study, an intelligent engineering management and control system based on a microservice architecture was applied to a super long-span valve hall project in Southeast China. The relevant information of the project are as follows: the length, span and height of the high-end valve hall are 89.5, 86, and 35.5 m, respectively and those of the low-end valve hall are 80.5, 68.5, and 22.8 m, respectively. The minimum span of the valve hall was 67 m, and the maximum span was 89.5 m. A photograph of the project site is shown in Fig 10.

**Fig 11:**
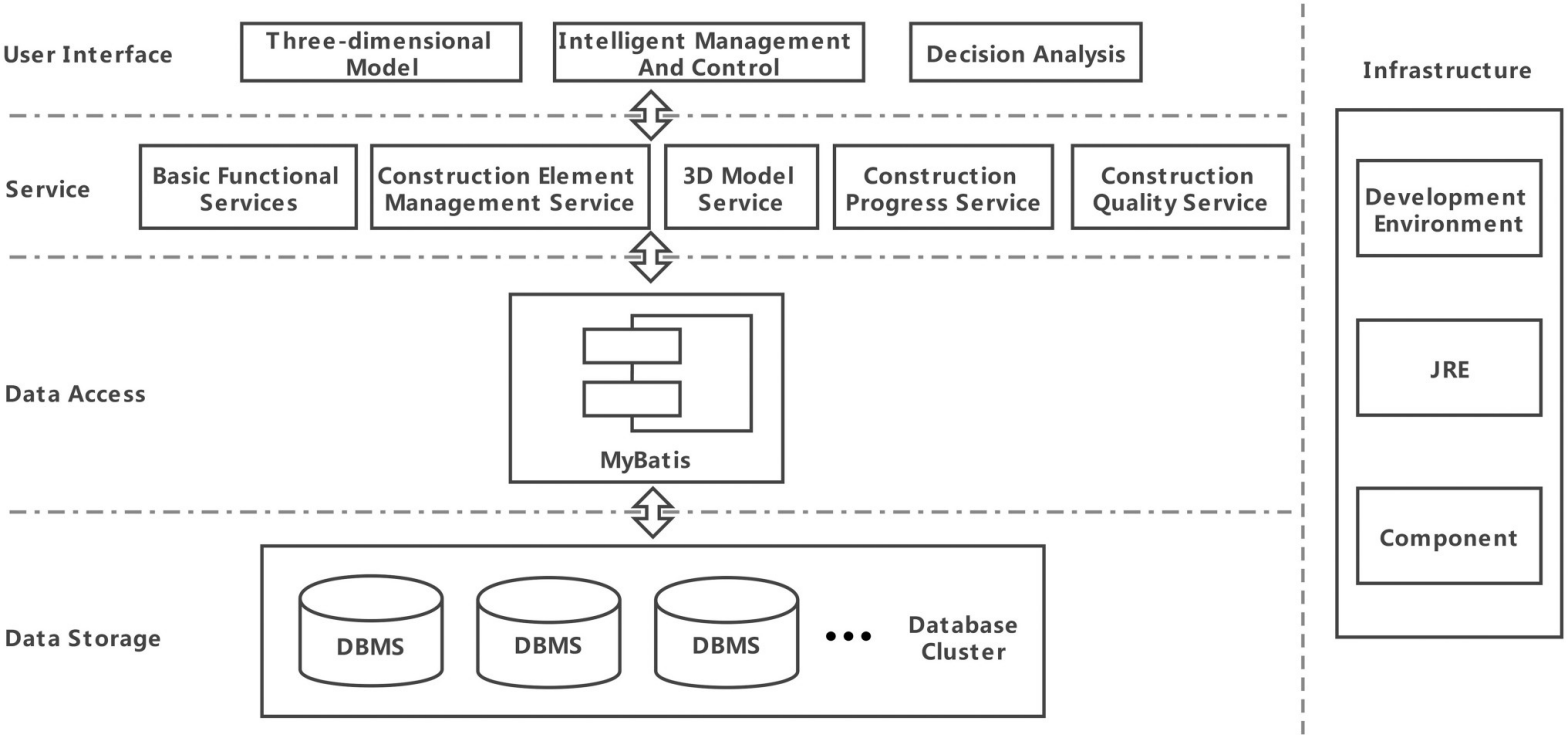
System architecture diagram.

**Fig 12:**
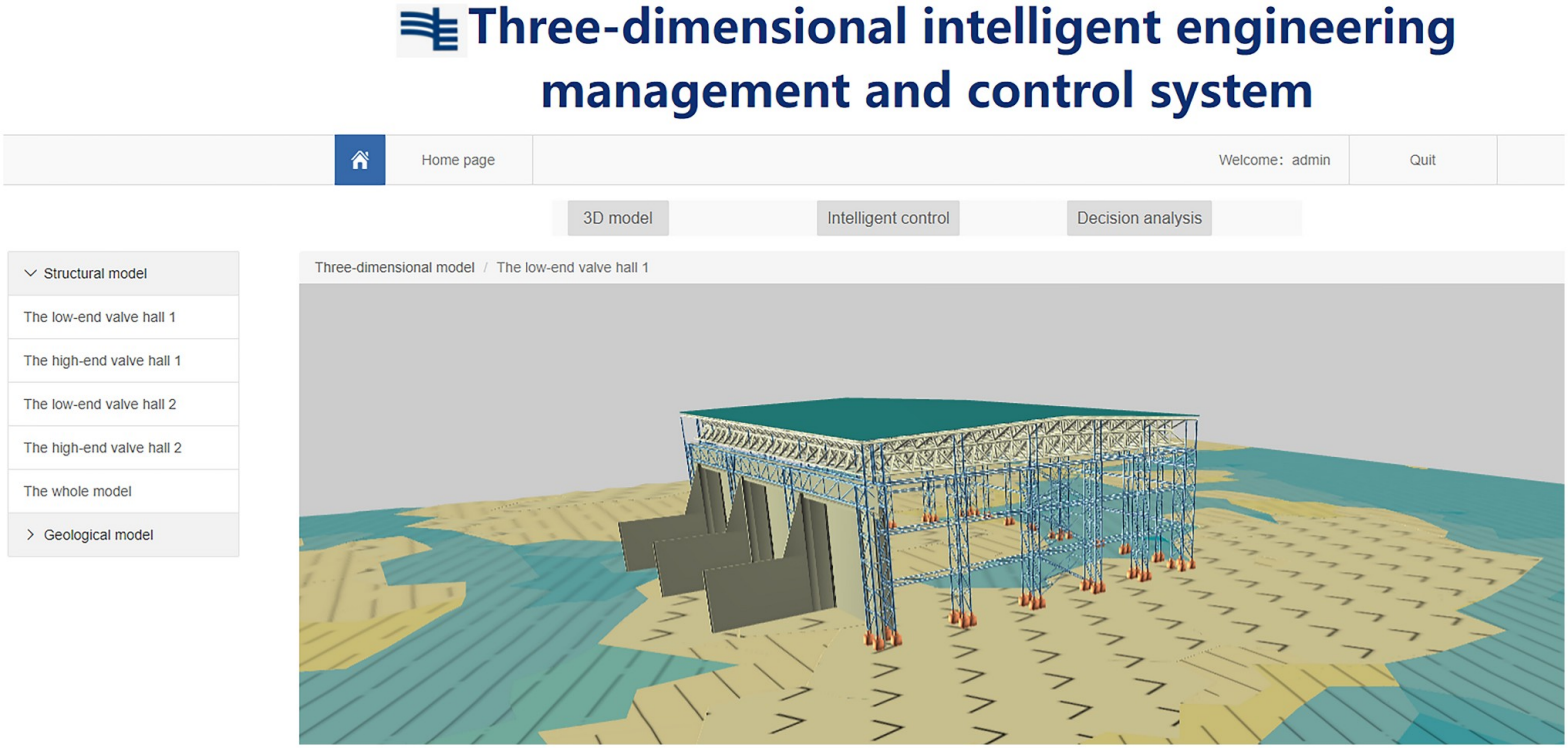
System home page.

At present, the system includes five services: a basic function service, construction element management service, 3D model service, construction progress service and construction quality service. The overall architecture of the system is shown in Fig 11, and the home page of the system is shown in Fig 12.

**Fig 13:**
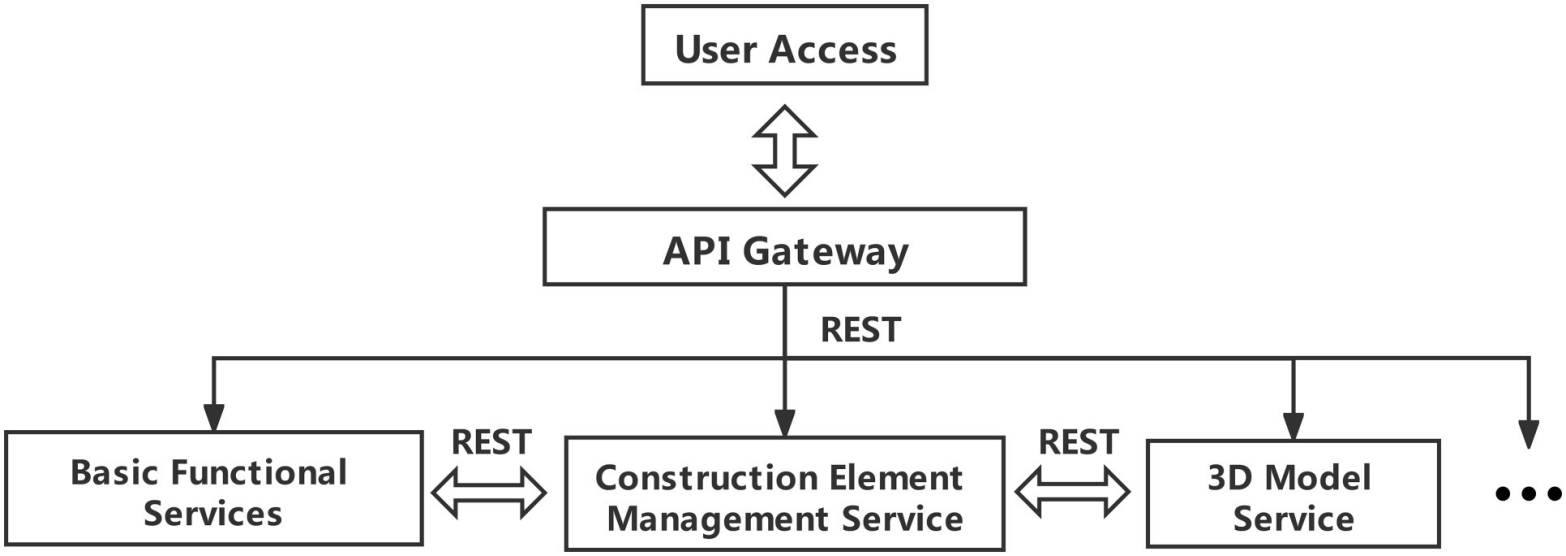
Communication between services.

The system uses Application Programming Interface Gateway (API Gateway) to provide a unified service portal for the whole system, aggregate background services, and provide API management functions such as security, filtering, flow control, etc. All services are independent processes run on different servers, so the communication between services is Inter-Process Communication (IPC). The system uses Representational State Transfer (REST) in the IPC solution, shown in Fig 13. REST is based on Hyper Text Transfer Protocol (HTTP), which makes the server implementation technology more flexible and has no program language restrictions. It can be called as long as the Software Development Kit (SDK) encapsulates HTTP.

The database cluster based on MSA is the base of the entire system, which provides data support for all microservices. The existing databases include user and permission databases, engineering design databases, numerical simulation databases, construction information databases, 3D model databases and monitoring databases. Based on the microservice architecture, database clusters can add new professional or type data through cluster expansion, without affecting the original data structure.

**Fig 14:**
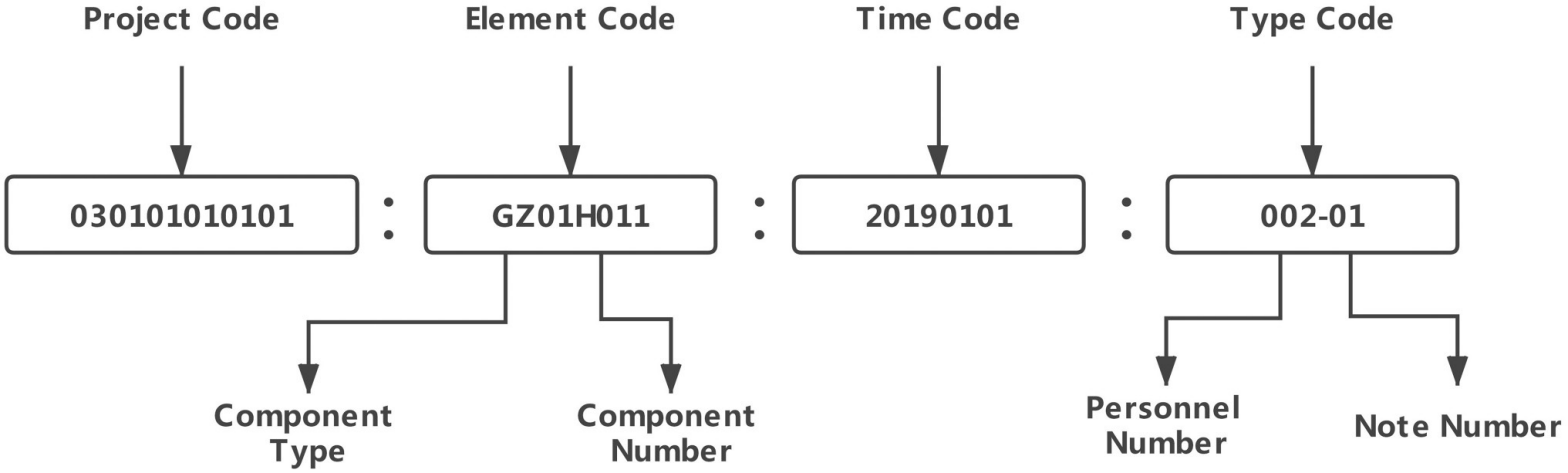
Information classification and coding system.

Basic function services provide the basic functions of the platform, such as user registration, login, exit, and permission control. Based on information classification and coding systems, the construction element management service is an application service for managing project lifecycle data. This service provides an independent work management environment for multisource heterogeneous engineering data and maintains massive amounts of data. In addition, to improve the transmission speed of a large-scale engineering data network, four layers of the coding system are set according to the actual situation of the project, corresponding to the project code, element code, time code and work type code, as shown in Fig 14.

By comparing the response time and server resource occupancy, it can be seen that on the premise of transmitting the same engineering information, engineering information classification and coding increase the speed of the network transmission of data and reduce the occupancy of server resources to realize the rapid querying and updating of large-scale engineering data. A comparison of the cloud server equipment and network bandwidth conditions is shown in Table 4, the experimental data is presented in Table 5 and the comparison information is presented in Table 6.

**Table 4:** Cloud server devices and network bandwidth conditions.

Name	Parameter
CPU and memory	2 cores, 8G
Hard drive capacity	80GB SSD
JDK	1.8
Network bandwidth peak	10 Mbps

**Table 5:** Comparison information.

Number	Type	Server memory footprint (bytes)	Network transmission Time (ms)
1	Form	256	280
2	Form	256	212
3	Form	256	256
4	Form	256	300
5	Form	256	232
6	Coding format	12	120
7	Coding format	12	112
8	Coding format	12	120
9	Coding format	12	124
10	Coding format	12	124
11	JSON	12	802
12	JSON	12	712
13	JSON	12	756
14	JSON	12	734
15	JSON	12	716

**Table 6:** Comparison information.

	Form	Coding format	JSON
Average network transmission Time (ms)	16	12	12
Server memory footprint (bytes)	256	120	744

**Fig 15:**
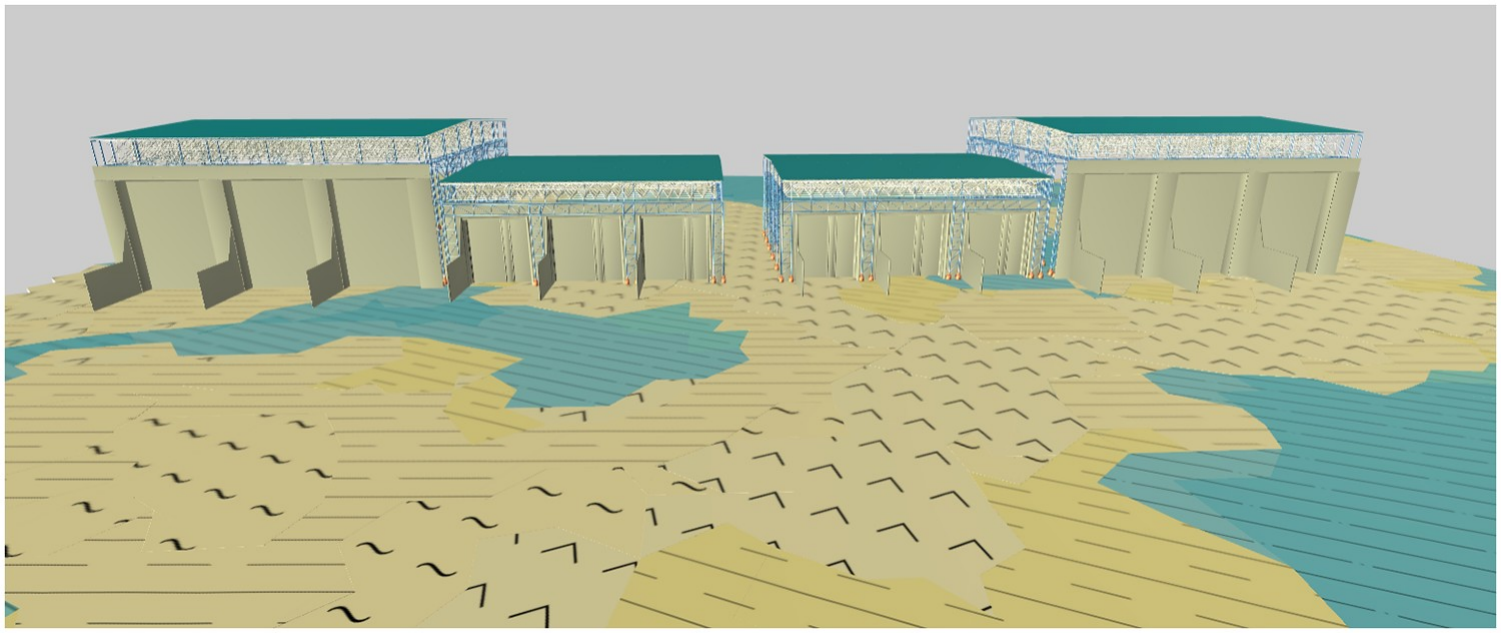
Integrated modeling of 3D engineering structure and geological environment.

**Fig 16:**
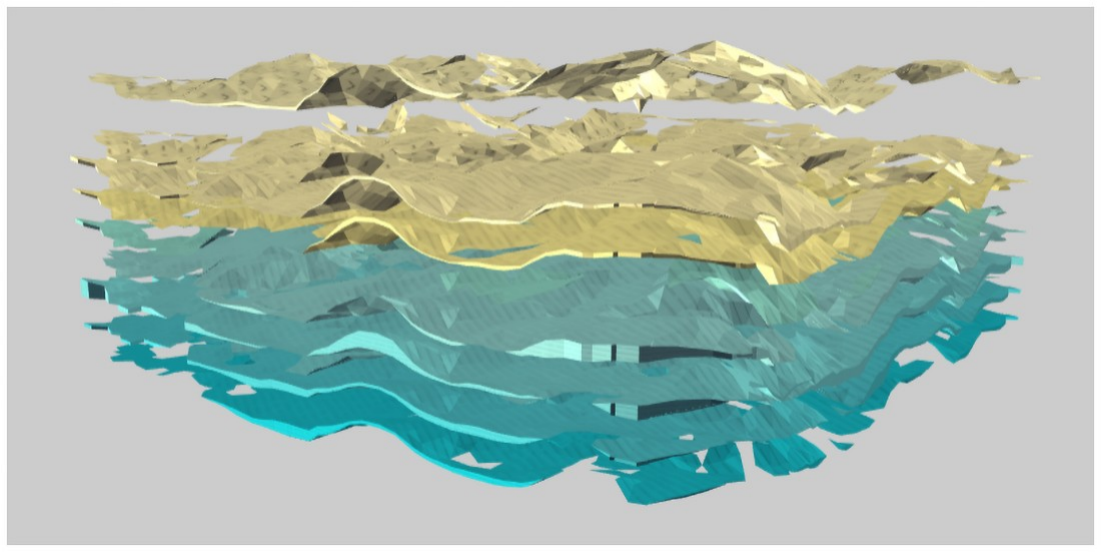
Stratigraphic display.

**Fig 17:**
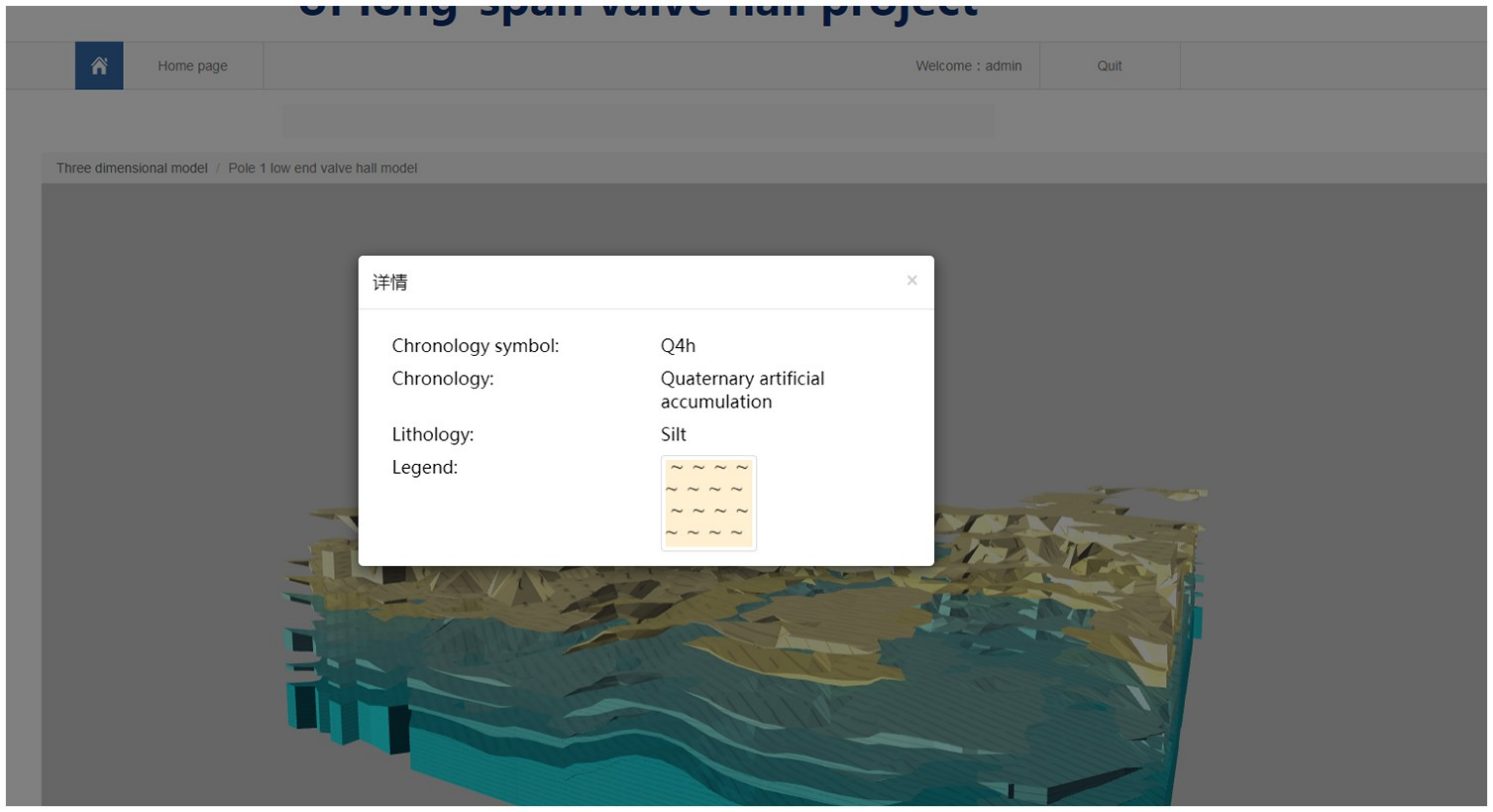
Stratum recognition.

**Fig 18:**
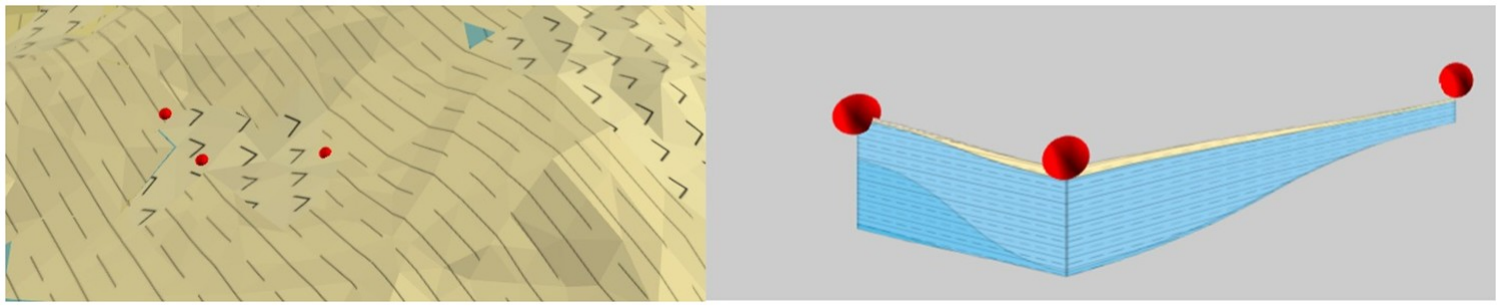
Stratum cutting.

**Fig 19:**
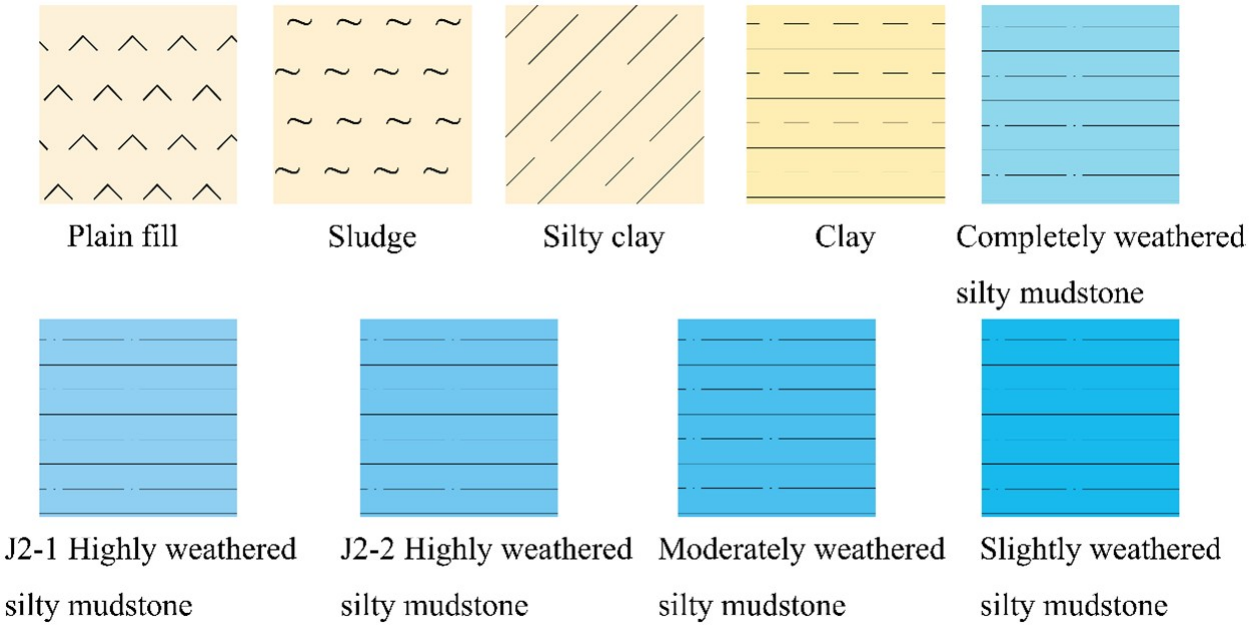
Lithologic legend of each layer.

The 3D model service realizes the integrated modeling of a 3D engineering structure and geological environment on the Internet by recording the topological relationship on the virtual contact surface, as shown in Fig 15. The service provides stratum stratification, stratum recognition and stratum cutting to strengthen the application of the 3D geological model. Figs 16–18 present the stratum stratification, stratigraphic stratum recognition, and stratum cutting, respectively. The lithologic legend of each layer is shown in Fig 19.

Using 3D model visualization, the construction progress service can accurately measure the construction status in real time through the client browser to ensure the orderly progress of each engineering link.

**Fig 20:**
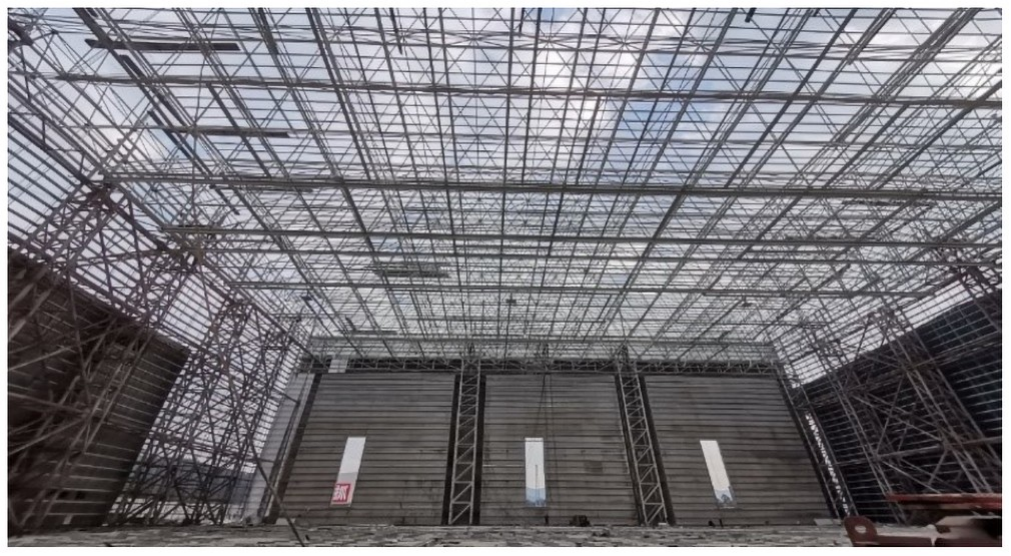
Image of the site construction during the lifting of the grid.

**Fig 21:**
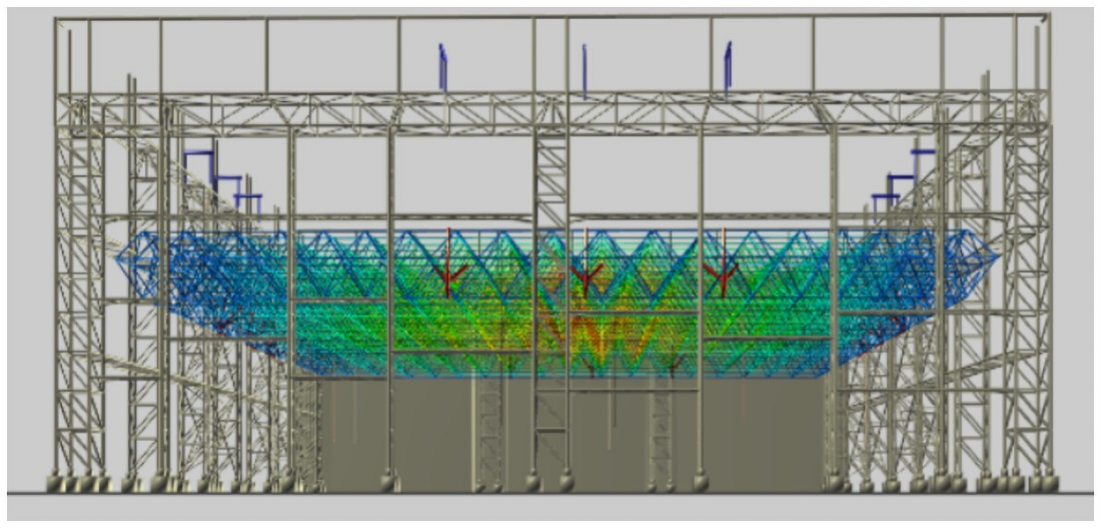
Visual presentation of numerical simulation on the web side.

**Fig 22:**
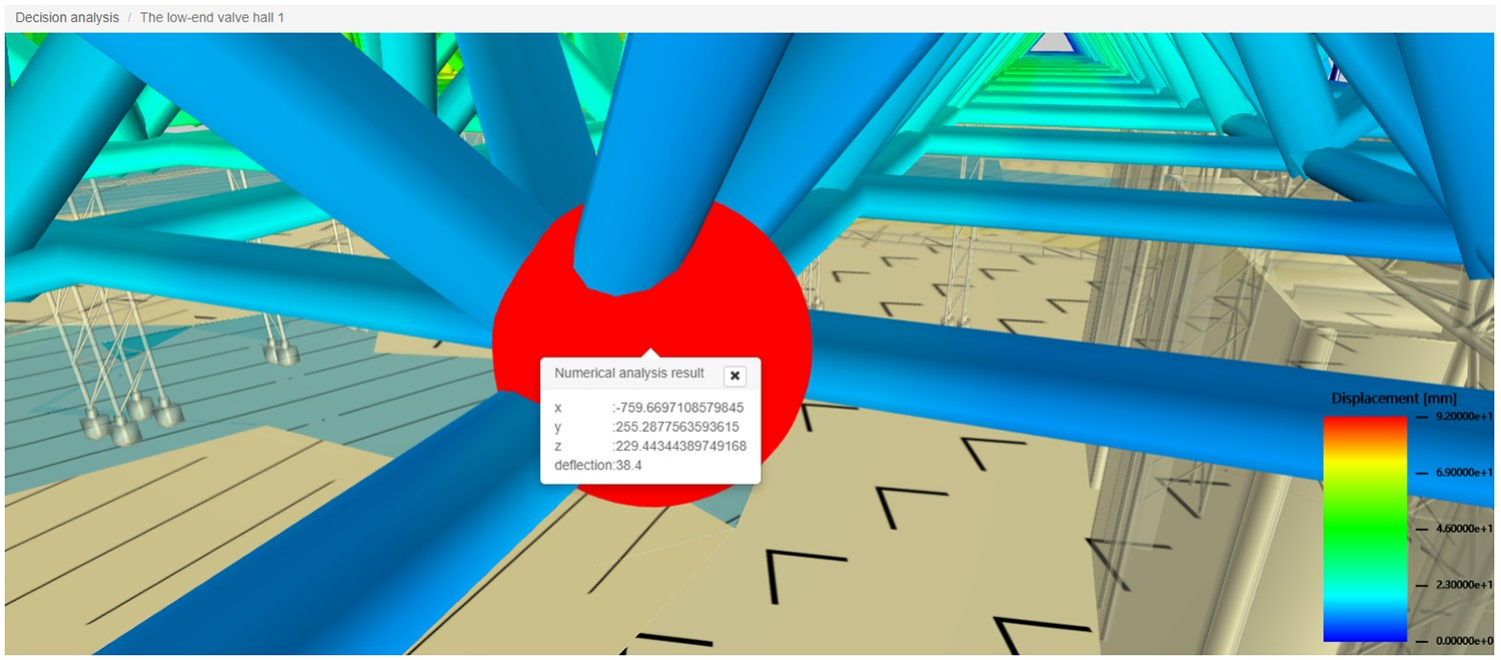
Key quality control point analysis.

The construction quality control service associates the numerical simulation data with the spatial node data of the three-dimensional model, realizes the visual display and query of the numerical analysis results on the webpage, and better serves the lifting of the long-span grid in the construction site. Fig 20 shows the site construction image of the lifting of the grid frame, and the three-dimensional visualization of the numerical simulation analysis is shown in Fig 21. The construction quality control service provides a comparison between the field-measured data and numerical simulation data of the key quality control points. If any quality problems are detected, then the causes are analyzed and corrected. They are then rechecked and compared until the quality requirements are met, as shown in Fig 22.

**Fig 23:**
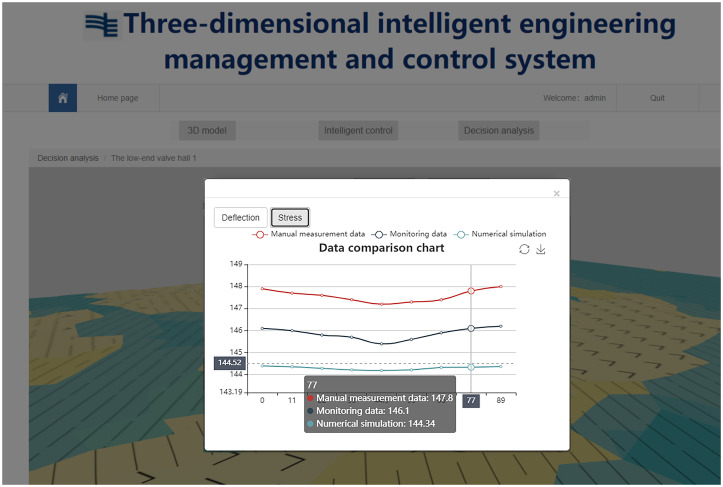
Stress along the center line of the long−span converter station steel structure.

**Fig 24:**
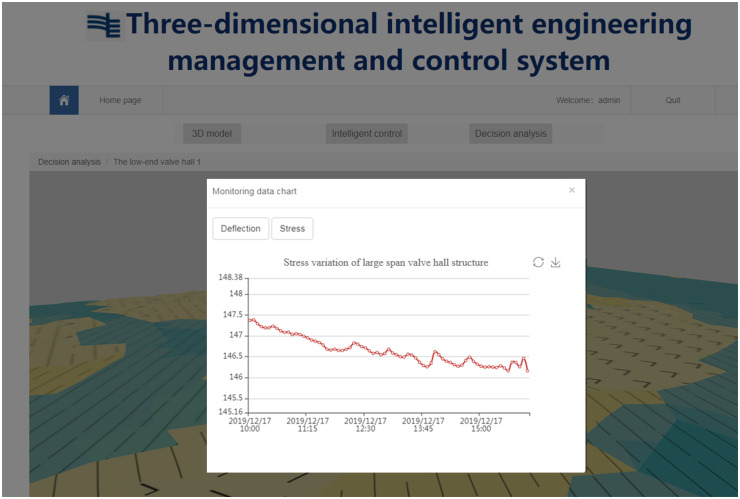
Monitoring data chart.

Furthermore, the system provides manual measurement data and monitoring data input services, online comparison of real-time monitoring data, manual measurement data and numerical simulation data through a web browser, which is convenient for relevant personnel to analyze the construction quality and safety, and serves the construction management of large-span steel structures. The maximum stress values identified from the numerical simulation, manual measurement, and automatic monitoring were all located at both ends of the converter station steel structure and gradually decrease towards the center, while the overall stress changed little, as shown in Fig 23. In the process of using the system, the real-time monitoring data are transmitted to the database, as shown in Fig 24, and the monitoring, calculation and analysis are integrated to synchronously generate the visualization of deflection and stress changes, truly reflect the on-site lifting situation. The data are from the monitoring results of the project site. When the mechanical properties of the long-span converter station steel structure change suddenly, it can be detected quickly, allowing for real time warning and response. This system overcomes the problems of complex operations, large amounts of data, low efficiency, and difficulty of management and control in the traditional long-span converter station steel structure hoisting monitoring method.

## 4 Conclusion

The management and application of multidisciplinary data in the large-span valve hall project proposed in this study effectively reduced the coupling of various types of engineering data through engineering database clusters and engineering information classification coding systems and realized the efficient management and application of these data. Integrated modeling of engineering structures and geological environments was proposed to establish the topological association between the valve hall engineering structure and geological environment, without increasing the amount of model data required. Three-dimensional intelligent control of the entire construction process of the super large-span valve hall project was proposed, and the real-time and accurate simulation and control of the construction process of the project was realized through WebGL technology.

By integrating the above-mentioned methods, a three-dimensional intelligent engineering management and control system based on MSA was applied to a super long-span valve hall project in Southeast China. The application showed that the engineering management and control system can quickly update, query and analyze the whole-stage information data of the valve hall project on the Internet, simplify the complex structure system of the super long-span valve hall project in the construction process, accurately measure the construction progress and control the construction quality, and ensure the smooth progress of the project construction. Simultaneously, each functional module maintained a low degree of coupling to improve the scalability of the system.

The application proposed in this study showed that the three-dimensional intelligent engineering management and control system for the construction of a long-span valve hall project based on a microservice architecture was reasonable and reliable, which can provide better support for "smart engineering" construction and promote the transformation from traditional construction management to cloud service intelligent construction management.
